# The Diversity and Geographic Distribution of Cultivable *Bacillus*-Like Bacteria Across Black Soils of Northeast China

**DOI:** 10.3389/fmicb.2019.01424

**Published:** 2019-06-21

**Authors:** Junjie Liu, Xiao Cui, Zhuxiu Liu, Zhaokui Guo, Zhenhua Yu, Qin Yao, Yueyu Sui, Jian Jin, Xiaobing Liu, Guanghua Wang

**Affiliations:** ^1^Key Laboratory of Mollisols Agroecology, Northeast Institute of Geography and Agroecology, Chinese Academy of Sciences, Harbin, China; ^2^School of Life Sciences and Technology, Mudanjiang Normal University, Mudanjiang, China; ^3^University of Chinese Academy of Sciences, Beijing, China; ^4^Institute of Tobacco Science, Heilongjiang Branch, China National Tobacco Corporation, Harbin, China

**Keywords:** *Bacillus*, biogeographic distribution, principal coordinates analysis, Illumina MiSeq sequencing, Mollisols

## Abstract

*Bacillus*-like species are gram-positive bacteria that are ubiquitous in soils. Many of *Bacillus*-like bacteria are demonstrated as beneficial microbes widely used in industry and agriculture. However, the knowledge related to their diversity and distribution patterns in soils is still rudimentary. In this study, we developed a combined research method of using culture-dependent and high-throughput sequencing to investigate the composition and diversity of cultivable *Bacillus*-like bacterial communities across 26 soil samples obtained from the black soil zone in northeast China. Nearly all bacterial 16S rDNA sequences were classified into the order Bacillales. Fifteen genera were detected, with *Bacillus*, *Paenibacillus*, and *Brevibacillus* being the three most abundant genera. Although more than 2,000 OTUs were obtained across all samples, 33 OTUs were confirmed as the abundant species with a relative abundance over 5% in at least one sample. Pairwise analysis showed that the diversity of *Bacillus*-like bacterial communities were significantly and positively correlated with soil total carbon contents and soil sampling latitudes, which suggests that a latitudinal gradient diversity of *Bacillus*-like bacterial communities exists in the black soil zone. The principal coordinates analysis revealed that the *Bacillus*-like bacterial communities were remarkably affected by soil sampling latitudes and soil total carbon content. In general, this study demonstrated that a distinct biogeographic distribution pattern of cultivable *Bacillus*-like bacterial communities existed in the black soil zone, which emphasizes that the strategy of local isolation and application of beneficial *Bacillus*-like strains is rather important in black soil agriculture development.

## Introduction

*Bacillus*-like bacteria (phylum Firmicutes; class Bacilli; order Bacillales), are a group of Gram-positive bacteria with low G+C% content (Ciccarelli et al., 2006; Wu et al., 2009). The most distinguishing features of *Bacillus*-like bacteria are that they can form endospores within cells that provide high resistance to radiation, desiccation, UV light, heat and chemicals, allowing these bacteria to survive under adverse conditions for an extended at a dormant stage (Garbeva et al., 2003; Mandic-Mulec et al., 2016). *Bacillus*-like bacteria are ubiquitous in nature, and many species have been isolated from diverse environments such as freshwater, saline water, soils, plants, animals, and air (Nicholson, 2002; Gardener, 2004), as well as from clean rooms in the Kennedy Space Center (Vaishampayan et al., 2010), and a vaccine-producing company (Seiler et al., 2013). *Bacillus*-like bacteria are one of the important components of the soil microbial community, and are often detected at a relatively high adundance in extreme environments, such as acidic soils (Felske et al., 1998), saline-alkali soils (Liszka et al., 2012), and desert soils (Felske et al., 2003), indicating that *Bacillus*-like bacteria play important roles in these soils.

Many species of *Bacillus*-like bacteria have a wide range of applications in bioenzyme production, biodefense, biofuel production, and bioremediation of toxic organic compounds (Kalia et al., 1994; Sonakya et al., 2001; Porwal et al., 2008), biofertilizer and biocontrol (Fira et al., 2018; Li et al., 2019). Thus, the researches related to the isolation, diversity and biogeographical distribution of *Bacillus*-like bacteria in soils has often been reported recently. For example, Ge et al. (2016) reported the distribution and diversity of cultivable *Bacillus*-like species in soils of Mount Wuyi, China, and they found that the diversity of *Bacillus*-like species varied among locations. The isolating frequency of some species had a significant correlation with altitude. Zhang et al. (2003) detected the diversity of cultivable *Bacillus*-like bacteria in four ecosystems in the red soil region across southern China, and they found that the diversity decreased in the order of forestland > upland >paddy field > eroded land. These findings indicated that the *Bacillus*-like bacteria in soils are not randomly distributed, and that a certain environmental factor might be the behind drivers for their distribution. In contrast, Liu et al. (2016) isolated 136 species of *Bacillus*-like bacteria from 20 soils in Taiwan, and no correlation in the species distribution among the sampling sites was observed.

Black soils, which are classified as dark Chernozems and referred to as Mollisols, are one of the most important soil resources for crop production in China (Liu et al., 2012). Black soils are primarily distributed in a long and narrow area called the black soil zone, which is approximately 900 km from the north to the south and 300 km from the east to the west and stretches across the three provinces of Heilongjiang, Jilin and Liaoning in northeast China. We collected 26 soil samples from arable farmlands across the black soil zone in 2014, and bacterial communities were shown to feature a distinct geographical distribution across the black soil zone using the high-throughput sequencing (HTS) method (Liu et al., 2014). In that study, the relative abundances of the phylum Firmicutes ranged from 0.18 to 12.12% with an average value of 1.44% across all soil samples, and the distribution pattern of this phylum was not analyzed (Liu et al., 2014).

Given that most of *Bacillus*-like bacteria are cultivable (Seldin et al., 1998; McSpadden Gardener, 2014), and most of studies investigating the distribution of *Bacillus*-like bacteria in soils are based on the isolation method, and the isolated strains were identified by 16S rRNA gene sequencing one by one (Liu et al., 2016, 2017; Mukhtar et al., 2018). This protocol is a laborious and time-consuming, which restricts us to investigate the diversity of *Bacillus*-like bacteria with high efficiency. In this study, we designed a research method of using culture-dependent and HTS methods to analyze the diversity and distribution patterns of cultivable *Bacillus*-like bacteria across black soil zone of northeast China.

## Materials and Methods

### Soil Samples

In this study, 26 soil samples (0–20 cm) were collected across the black soil zone of northeast China based on the database for Scientific Data Center of Northeast Black Soil^1^ (Supplementary Figure S1). The methods of soil sampling and physicochemical property determination were described in our previous studies (Liu et al., 2014). Since *Bacillus*-like bacteria formed endospores that can resist to various stresses such as dryness, high temperatures, etc., the soils used for this study were from archived room-temperature dried soils that were kept in glass bottles. The soil physicochemical properties are listed in Supplementary Table S1.

### *Bacillus-*Like Bacterial Cell Incubation

Five grams of each dry soil sample was added into a conical flask containing 45 mL of sterilized water. The flask was treated by an ultrasound machine (JAC500N, Suzhou, China) at 60 kHz with a power density of 0.3 W/cm^2^ for 60 s, and then shaken at 200 rpm for 45 min. After that, the flask was put on an experimental table and left for 20 min. Then 1.5 mL of the supernatant of soil solution was pipetted into a sterilized centrifuge tube, and the tube was incubated in a water bath machine at 85°C for 50 min. Subsequently, the solution was serially diluted 1,000 times, and 200 μL of the final solution was spread on the surface of 1/2 strength NA-nutrient agar in a 90 cm petri dish. Each soil solution was spread on three dishes. After the dishes were inversely incubated at 15°C for 10 days, 10 mL of sterilized water was added into each plate, and the bacterial cells were scraped off using a sterilized glass rod to generate a bacterial suspension as reported in our previous study (Xu et al., 2009). Then, the cell suspensions from three dishes for each soil were mixed and transferred into a 50 mL centrifuge tube, and the tube was centrifuged at 10,000 rpm for 10 min to collect the bacterial pellet. The pellet was stored at -80°C for DNA extraction.

### Bacterial Cell DNA Extraction

DNA from the bacterial pellet was extracted using a Fast DNA^®^ Spin Kit for Soil (MP Biomedicals, United States) according to the manufacturer’s instructions. Extracted DNA was diluted in TE buffer (10 mM Tris–HCl, 1 mM EDTA, pH 8.0), and the quality of the DNA solution was checked using a NanoDrop 2000 Spectrophotometer (Thermo Fisher Scientific, United States). Extracted DNA was stored at -20°C for downstream analysis.

### Illumina MiSeq Sequencing

DNA extracted from each sample was used as a template for PCR amplification by the primers F515/R907 (Biddle et al., 2008). The forward and revised primers were modified with a unique 8 nt barcode at the 5′ end to distinguish the soil sample. A 25 μL PCR reaction mixture contained 23 μL of Platinum PCR SuperMix (TransGen Biotech Co., Ltd., Beijing, China), 0.5 μL of each primer (10 μM), and 1.0 μL of DNA template (10 ng). Amplification was performed under the following conditions: initial denaturation at 95°C for 3 min, followed by 30 cycles (95°C for 1 min, 63°C for 1 min, 70°C for 1 min), and extension at 72°C for 5 min. Each DNA sample was amplified in triplicate, with the PCR products run on a 1.5% agarose gel and then purified using the Agarose Gel DNA purification kit (Takara, Dalian, China). The purified PCR products were normalized to equimolar amounts, combined into one pooled sample, and then sequenced (2 × 300) using an Illumina MiSeq platform at the Majorbio Biotechnology (Shanghai, China).

### Processing of Sequencing Data

The process of sequencing data was conducted in a similar manner to those in Lauber et al. (2008), and our recent studies (Liu et al., 2018, 2019). Briefly, the raw FASTAQ data were processed using QIIME Pipeline Version 1.9.0 (Caporaso et al., 2010). Forward and reverse reads were joined using FLASH software with a minimum of 10 bp overlap (Magoč and Salzberg, 2011). Low-quality sequences that were shorter than 300 bp in length, had a mean quality score of less than 25, or that contained ambiguous bases, were discarded for further analysis. The chimeras of trimmed sequences were filtered and removed with the UCHIME algorithm (Edgar et al., 2011) in the USEARCH tool against the “RDP Gold” database. The high-quality sequences were assigned using the UPARSE pipeline^2^ at a 97% similarity threshold to generate OTUs (operational taxonomic units). The taxonomic classification of each representative sequence was aligned with the Python Nearest Alignment Space Termination (PyNAST) tool (DeSantis et al., 2006), and a neighbor-joining tree was constructed using FastTree within the QIIME Pipeline according to the regulated procedures (Price et al., 2009). The taxonomic identity of each 16S rRNA gene was performed using the RDP classifier with a confidence threshold of 0.80^3^ (Cole et al., 2005). After the taxonomies were assigned, the sequences that did not match those of *Bacillus*-like bacteria were discarded from the database. Then, the new data, excluding non-*Bacillus*-like bacteria, were reanalyzed from the OTUs generated. The raw reads of the 16S rRNA have been submitted to the National Center for Biotechnology Information (NCBI) Sequence Read Archive (SRA) under the accession number PRJNA525189.

### Statistical Analysis

To avoid the effects of uneven sequence bias, a randomly selected subset of 27,900 sequences of each sample was subjected to for alpha diversity and beta diversity analyses. Alpha diversity of phylotype richness (OTUs) and phylogenetic diversity (PD) (Faith, 1992) were conducted with the QIIME Pipeline and fitted against soil carbon content and latitude in a linear regression using SPSS 18.0 for Windows. Alpha diversity was displayed with ArcGIS software (Version 10.2.1), and the goodness of fit was evaluated by adjusted *R^2^* and *P*-values. The sequences of major OTUs (over 5% in at least one sample) were aligned with taxonomy-determined sequences obtained from the NCBI database using ClustalX 1.81 (Thompson et al., 1997), and a neighbor-joining phylogentic tree of *Bacillus*-like bacterial communities was constructed with Molecular Evolutionary Genetic Analysis software (MEGA 7.0) with a Kimura 2-parameter model at 1000-fold bootstrap support (Kumar et al., 2016).

To compare the differences of *Bacillus*-like bacterial communities, based on a weighted and unweighted UniFrac distance matrix, principal coordinate analysis (PCoA) (Gower, 1966) was used to analyze the beta-diversity with the “ape” library in the R environment (version 3.2.5) (R Development Core Team, 2010). The linear relationships between PCoA scores and soil total carbon content (TC), and soil pH value were determined with SPSS 18.0 for Windows. Meanwhile, based on the Bray-Curtis distance, a cluster analysis of the *Bacillus*-like bacterial communities was performed using the “vegan” packages in the R environment. Furthermore, a heatmap analysis was conducted using the “pheatmap” package and displayed with the “ggplot2” package in the R environment to reveal the changes of major OTUs among different soils.

## Results

### Distribution of *Bacillus*-Like Bacteria

In total, 1,024,205 high-quality quality sequences were obtained from all 26 soil samples, with 27,981-55,133 sequences per sample (mean 39, 392). Of these sequences, 99.88% could be classified as phylum_Firmicutes, class_Bacilli, order_Bacillales by classifier alignment using the RDP database. The read lengths ranged from 372 to 520 bp with a mean of 376 bp. When grouped at the 97% similarity level, there were 2, 027 different phylotypes across all soil samples, with an average of 523 phylotypes per sample.

Five families were detected in this study. Among them, Bacillaceae was the most abundant, with an average relative abundance of 53.99% (ranging from 17.79 to 96.75%), followed by Paenibacillaceae (with an average relative abundance of 34.10% and ranging from 0.80 to 81.06%), and Planococcaceae (with an average relative abundance of 11.71% and ranging from 0.20 to 59.426%); the sequences belonging to families of Staphylococcaceae and Thermoactinomycetaceae were randomly detected in some soils in very small amounts (Supplementary Table S2).

**FIGURE 1:**
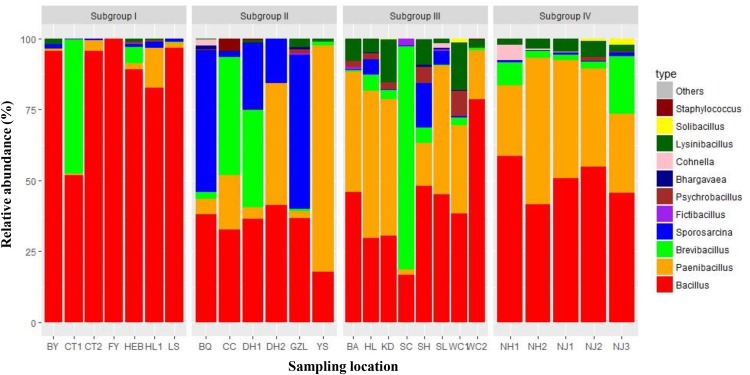
Relative abundances of the dominant *Bacillus*-like bacterial groups at genus level. Relative abundances are based on the proportional frequencies of the *Bacillus*-like bacterial sequences that could be classified.

At the genus level, at least 15 genera were detected. Among them, *Bacillus*, *Paenibacillus*, and *Brevibacillus* were the three most abundant genera, with average relative abundances of 53.82, 23.50, and 10.22% across all samples, respectively. *Sporosarcina* and *Lysinibacillus* were the fourth and fifth most abundant genera with average relative abundances of 7.05 and 3.27%, respectively. Other genera, such as *Psychrobacillus*, *Cohnella*, *Sporosarcina*, and *Staphylococcus*, were sporadically detected with much lower average relative abundances (less than 1% across all soil samples) (Supplementary Table S2). Figure 1 shows the distribution patterns of the obtained Bacillales sequences at the genus level according the grouping analysis described below. The samples in subgroup I contained a relatively higher abundance of *Bacillus*, with the exception of CT1, which contained 47.48% of *Brevibacillus*; Samples in subgroup II contained relatively higher abundances of *Sporosarcina* than other subgroups, with the exception of YS, which contained 79.87% of *Paenibacillus*; In contrast, samples in subgroups III had relatively higher amounts of *Lysinibacillus* and *Paenibacillu*s, and subgroup IV contained relatively higher amounts of *Paenibacillus*.

**FIGURE 2:**
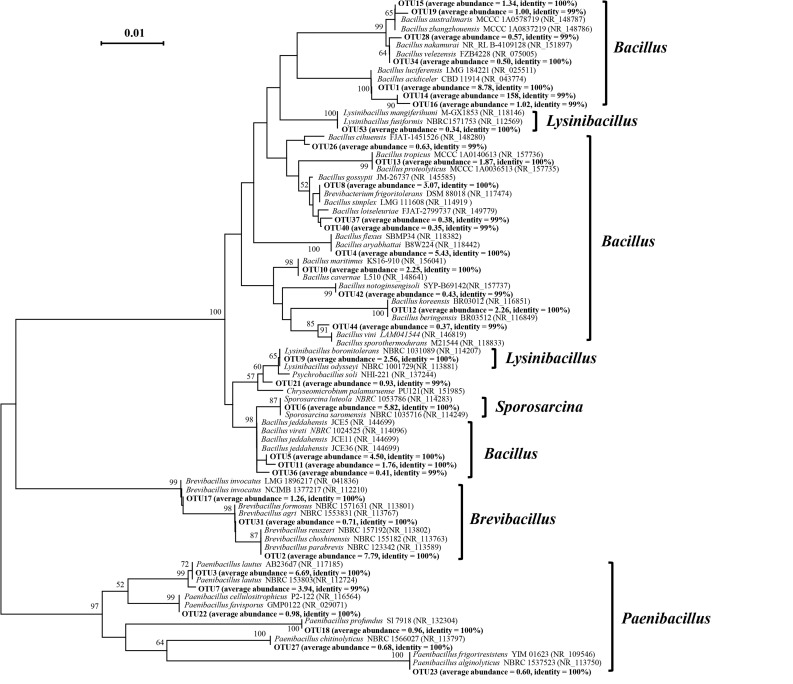
Neighbor-joining phylogenetic tree showing the relationships of the representative sequences of 33 abundant OTUs of *Bacillus*-like bacterial communities obtained from the black soils. The numbers in the parentheses behind each OTU represents the average abundance of the OTU and the identity between the OTUs and the closest reference bacteria, and the numbers in the parentheses of reference bacterial strains indicate the accession numbers in the NCBI website.

More than 2,000 OTUs were detected in this study; however, the majority of them were observed with a very low abundance. When considering the OTU threshold of having a relative abundance of over 5% in at least one sample, there were 33 OTUs observed in this study. Figure 2 shows the phylogenetic tree based on the average relative abundance of OTUs, and the similarity with the closest reference strains. Among them, 20, 6, 3, 3, and 1 OTUs were grouped into genera of *Bacillus*, *Paenibacillus*, *Brevibacillus*, *Lysinibacillus*, and *Sporosarcina*, respectively. In addition, the distribution pattern of these OTUs in different soils varied significantly. For example, OTU4, 3, 1, 8, 2, 5, 9, 10, 11, and 6 were observed in most soil samples (>20 samples), while OTU12, 18, 27, 23, and 53 were only sporadically detected in fewer than 10 soil samples (Supplementary Figure S2).

### Alpha Diversity of Cultivable *Bacillus*-Like Bacterial Communities

**FIGURE 3:**
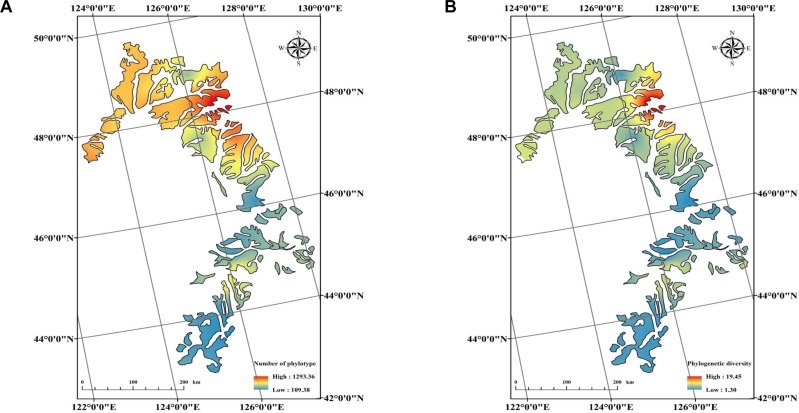
Maps showing alpha diversity of phylotype richness (OTUs) **(A)**, and phylogenetic diversity **(B)** in 26 samples across the black soil zone.

**FIGURE 4:**
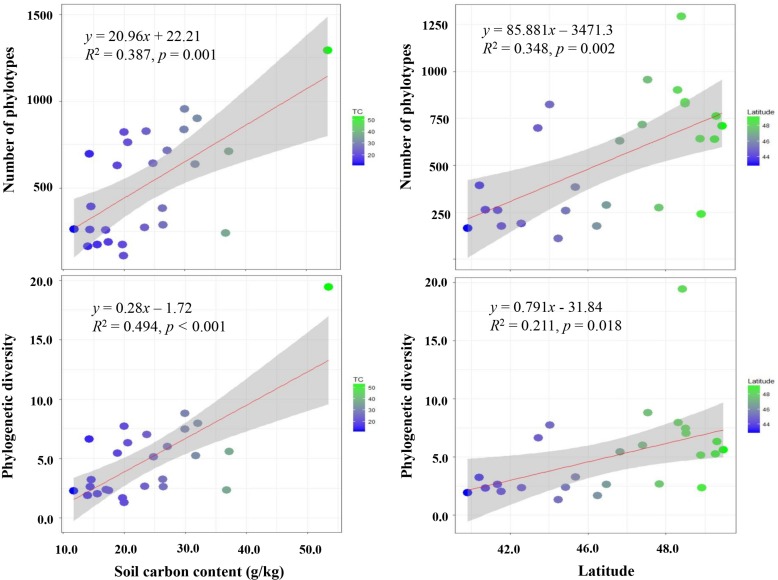
The linear relationships between the phylotype richness (OTUs), phylogenetic diversity (PD) and soil carbon content and latitudes.

To compare the cultivable *Bacillus*-like bacterial community diversity among all soils, a survey at the same level of 27,900 sequences that were randomly selected from each sample sequencing library was performed. Alpha diversity was highly variable and associated with PD (ranging from 1.31 to 19.43) and phylotype richness (ranging from 110 to 1294) among 26 soils, with samples FY and BA having the lowest and the highest diversity, respectively. The maps of alpha diversity clearly showed that phylotype richness and PD varied along a color gradient across the black soil zones, with soils from higher latitudes having more phylotype richness and PD (Figure 3). The result was further confirmed by pairwise analysis which indicated that both PD and phylotype richness were significantly and positively correlated with soil total carbon contents (TC) and soil sampling latitudes (Figure 4).

### Beta Diversity of Cultured *Bacillus*-Like Bacterial Communities

**FIGURE 5:**
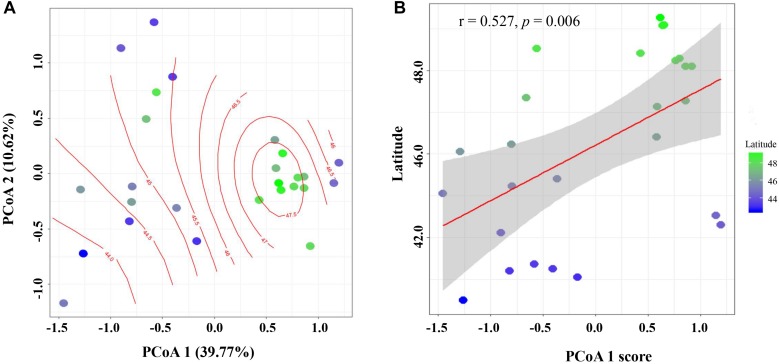
Principal coordinate analysis of *Bacillus*-like bacterial communities based on the unweighted pairwise UniFrac community distances between sites **(A)**. The linear relationships between the PCoA1 score and latitude **(B)**.

**FIGURE 6:**
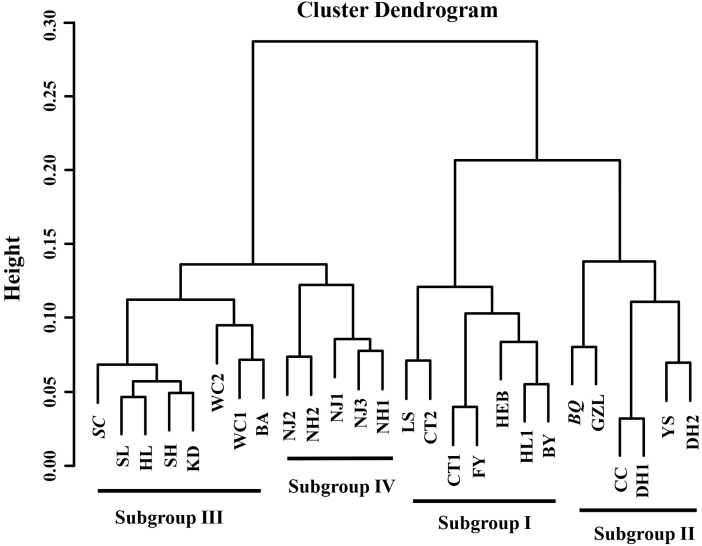
Cluster analysis of *Bacillus*-like bacterial communities based on the Bray-Curtis distance matrix. The letters represent the sample location across the black soil zone. Such as CT1 and CT2 represent the locations of Cangtu 1 and Cangtu 2 from Liaoning Province, respectively, and additional detailed information for each sample location was shown in Supplementary Table S1.

The principal coordinates analysis (PCoA) plot of cultivable *Bacillus*-like bacterial communities based on the weighted (Supplementary Figure S3) and unweighted Unifrac distance (Figure 5) matrix indicated that the community distribution across all soils were strongly influenced by sampling latitude, since both the score of weighted and unweighted PCoA1 was significantly and positively correlated with latitudes (weighted: *r* = 0.649, *P* < 0.001; unweighted: *r* = 0.527, *P* = 0.006), and TC (weighted: *r* = 0.601, *P* < 0.001; unweighted: *r* = 0.513, *P* = 0.007), respectively (Figure 5 and Supplementary Table S3). However, the communities had no relationship with soil pH, since the *P*-value between both PCoA1 and PCoA2 scores and pH was greater than 0.05 (Supplementary Table S3). Therefore, the *Bacillus*-like bacterial community structures obtained from soils of low latitudes were different from those of higher latitudes. All cultivable *Bacillus*-like bacterial communities were roughly clustered into four subgroups based on the Bray-Curtis dissimilarity distance (Figure 6). Group I consisted of seven soils from the southern and middle parts of the black soil zone, and their latitudes ranged from 42°50′N to 46°23′N. Group II consisted of six soils from southern parts of the black soil zone except for BQ, and sampling locations ranged from 43°26′N to 44°53′N. Groups III and IV contained eight and five soils mainly collected in the northern part of the black soil zone (with the exception of SC), with latitudes ranged from 46°41′N to 48°52′N and from 48°41′N to 49°26′N, respectively.

## Discussion

### About the Methods

Our previous study using the HTS method showed that the average relative abundance of Firmicutes across 26 black soils accounted for only 1.49% of the total sequences. Among these sequences, only five OTUs were observed with more than 100 sequences across all samples, and four and one of them belong to orders Clostridiales and Bacillales, respectively (Liu et al., 2014). Since very few of *Firmicutes* sequences were detected, the biogeographic distribution of Firmicutes in the black soils was not analyzed.

Given the crucial roles of *Bacillus*-like bacteria in agriculture and industry, the works of isolating the functional analysis of *Bacillus*-like bacteria is very important. However, prerequisite for this study, knowing the diversity of *Bacillus*-like bacteria in the natural environment is very important. In this study, we combined the culture-dependent and HTS methods to analyze the *Bacillus*-like bacteria, and the results indicated that nearly all sequences obtained from this study were classified into the order Bacillales, suggesting that this method is applicable to analyze cultivable *Bacillus*-like bacteria, avoiding of time-consuming one-by-one isolation. We confirmed that all the sequences obtained from this study were isolated from multiple bacterial growth on the plates, but not from contaminated cells of the inoculation soil suspension, since none of the sequences classified into Clostridiales, which are anaerobic and facultative anaerobic microbes for whom oxygen is toxic to them (Ferrer et al., 2013).

It is commonly known that the culture-independent methods reveal more diversity of microbial communities in soil than culture-dependent methods (Steven et al., 2007; He et al., 2008). However, for the specific groups, the cultivable method also has some advantages. For example, the cultivable bacteria with very low abundances in the soils and they are difficult to be detected through HTS, however, when the cultivable bacteria was grown on the plates, their number were increased and the DNA information of those bacteria was easily to be detected using molecular biological methods. In this study, we detected more than 2,000 OTUs of *Bacillus*-like bacteria on the plate, and majority of them were not observed by using the HTS method directly from soil samples (Liu et al., 2014), which suggested that the method of this study has its merits. Noticeably, although the dry soils were used for this study, the endospores formed by *Bacillus*-like bacteria guaranteed that the bacteria could survive when conditions were appropriate for their growth.

### Biogeographic Distribution of *Bacillus*-Like Bacteria

The biogeographic distribution of bacterial communities across the black soil region was reported in our previous study (Liu et al., 2014). Although the exact number of cultivable *Bacillus*-like bacteria in soils is unknown, the results of this study demonstrated that this kind of bacteria are also not randomly distributed and that there are certain rules or factors driving the distribution. First, we observed that the alpha diversity of *Bacillus*-like bacteria had a significant positive correlation with soil TC and sampling latitudes (Figure 4). This finding is inconsistent with the total bacterial community in the same soil samples, which showed that bacterial diversity was affected by soil pH and negatively related with sampling latitudes (Liu et al., 2014). This finding suggests that the higher of soil nutrition supports a higher diversity of soil cultivable *Bacillus*-like bacteria. Second, we observed that the beta diversity of cultivable *Bacillus*-like bacterial communities was strongly influenced by sampling latitude, which indicates that the biogeographic distribution of the cultivable *Bacillus*-like bacterial community exists across the black soil zone. This finding somewhat supports the results of Ge et al. (2016), who stated that the distribution of cultivable *Bacillus*-like species in soils of Mount Wuyi was closely correlated with the altitude, but it contrasts the results of Liu et al. (2016), who observed that the distribution of cultivable *Bacillus*-like species in Taiwan region had no relationship with sampling locations.

The reason for soil pH being the major soil factor driving the biogeographic distribution of the soil bacterial community was mainly ascribed into the phenomenon of the bacterial growth is very sensitivity to pH (Fierer and Jackson, 2006; Lauber et al., 2009). However, the *Bacillus*-like bacteria, they can grow in extreme conditions, even in the acidic soils (Felske et al., 1998) and saline-alkali soils (Liszka et al., 2012), which indicated that soil pH was not the dominant factor driving the cultivable *Bacillus*-like bacterial communities. Given that the soil TC content was closely positively related to sampling latitude in this study (*r* = 0.655, *P* < 0.001), the alpha and beta diversities of cultivable *Bacillus*-like communities are related to latitude (Figure 4), or, in another words, are also closely related to soil TC content (Supplementary Table S3). An incubation experiment by Watanabe et al. (2011) using ^13^C labeled glucose as a carbon source showed that ^13^C-enriched bacterial 16S rRNA gene clones were mainly belonged to *Bacillus* spp., *Paenibacillus* spp., and *Clostridium* spp. Thus, it seemed that soil nutrition, especially carbon source content, is highly related to the distribution of cultivable *Bacillus*-like bacteria, although this event was not revealed until now.

### Composition of Cultivable *Bacillus*-Like Bacteria

The traditional way to analyze the diversity of *Bacillus*-like bacteria is based on bacterial strain isolation and 16S rRNA gene identification (Maughan and Van der Auwera, 2011; Dheemana et al., 2017; Panosyan et al., 2018). Recently, the use of HTS surveying of *Bacillus*-like bacteria directly in soils and combining it with a one-by-one isolation method was also reported (Liu et al., 2017; Mukhtar et al., 2018). Those studies showed that more *Bacillus*-like bacterial genera were detected by HTS than by the culture-dependent method, which indicated that HTS was a more effective way to discover specific microbial diversity (Li et al., 2015). However, in this study, we detected more *Bacillus*-like bacterial genera by a culture-dependent method than by direct sequencing from soil samples. Higher diversity of *Bacillus*-like bacteria observed in this study may result from two reasons: one is *Bacillus*-like bacteria in soil are present in very low abundances, and the DNA information was covered by that of other bacteria when conducing HTS; the second reason is the traditional one-by-one isolation method may miss some information from *Bacillus*-like bacteria that grow in the medium but at low abundance.

Although a relatively high diversity of cultivable *Bacillus*-like bacteria inhabited in the black soils, only 33 OTUs were abundant OTUs with the relative abundance larger than 5% at least in one sample, which also inferred that the majority of cultivable *Bacillus*-like bacteria in soils were simplified. This finding is in consistent with results reported by Liu et al. (2016), who isolated 20 *Bacillus*-like species from 136 isolates from Taiwan soils, and also consistent with results from Ge et al. (2016), who obtained 42 species from 418 isolates from Mount Wuyi, but low than data observed by Liu et al. (2017), who identified 66 *Bacillus*-like species from 349 isolates from potato rhizosphere soils in Yili, Xinjiang, China.

Among 33 abundant OTUs, six OTUs were detected with an average relative abundance larger than 4% across all soil samples (Figure 2). OTU1 has 100% similarity to *Bacillus luciferensis* and *B. acidiceler*, and its relative abundance across all samples was 8.78%. *B. luciferensis* can bioremediate chromium pollution (Cheng and Li, 2009) and biocontrol of phytophthora blight in pepper (Kim et al., 2009). *B. acidiceler* was found with function of inhibit growth of *Phytophthora cinnamomi* through the production of volatile compounds (Méndez-Bravo et al., 2018). The average of relative abundance of OTU2 was 7.79%, and it has 100% similarity to *Brevibacillus reuszeri*, *B. choshinensis*, and *B. parabrevis*. The function of *B. reuszeri* and *B. parabrevis* was unclear, while, *B. choshinensis* has the ability to produce extracellular protein (D’Urzo et al., 2013). OTU3 has an average relative abundance of 6.69%, and it has 100% similarity to *Paenibacillus lautus*. *P. lautus* was detected to have the potential ability for cellulose degradation (Suman and Kumar, 2018). The average relative abundance of OTU6 was 5.82% across all samples; OTU6 has 100% similarity to *Sporosarcine luteola* and *S. saromensis*. *S. luteola* was a novel species isolated from the hopper surface of equipment used for sauce production in Japan (Tominaga et al., 2009). *S. saromensis* was reported has an ability of bioremediation of chromium pollution (Zhao et al., 2016). The average relative abundance of OTU4 was 5.41% across all samples, OTU4 is closely related to *Bacillus aryabhattai* and *B. flexus*, *B. aryabhattai* was identified with arsenic resistance and can promote rice seedling growth (Ghosh et al., 2018), and *B. flexus* has many functions in soils, such as nitrogen fixation (Yousuf et al., 2017), plant growth promotion (Wang et al., 2017), and PAHs degradation (Alrumman et al., 2016). OTU5 is the sixth most abundant bacteria in this study (relative abundance 4.50%), and it is closely related to *Bacillus jeddahensis* and *B. vireti*, *B. jeddahensis* was first isolated from a stool sample of a man living in Jeddah, Saudi Arabia. Recently *B. jeddahensis* JCE^T^ was fully sequenced, and it contains 4,654 protein-coding and 98 RNAs genes (Bittar et al., 2015). While *B. vireti* has many nitrogen-cycling related genes, and it was demonstrated to be a potent source and sink for nitric and nitrous oxide under high nitrate conditions (Mania et al., 2014). Through this analysis, we found that the majority of cultivable *Bacillus*-like bacteria in the black soils are the functional or beneficial bacteria, and the isolation, purification and application of these bacteria will be beneficial for maintaining soil health and promoting crop products. The implication of this study is that, we demonstrate that cultivable *Bacillus*-like bacteria are distinctly biogeographically distributed across black soil regions; therefore, the local isolation strategy is critical for the application of *Bacillus*-like bacteria in the soils.

## Conclusion

In conclusion, this is the first comprehensive study using a combination of the culture-dependent and HTS methods to investigate the cultivable *Bacillus*-like bacterial community distribution pattern in the black soil zone of northeast China. Our results showed that the diversity of *Bacillus*-like bacteria was positively correlated with the latitudes of sampling locations, and this finding differed from that of the total bacterial communities (Liu et al., 2014), which showed that a higher bacterial diversity was detected at lower latitudes in the same soil samples. Compared with whole bacterial communities, another inconsistency is that the environmental factors controlling the distribution pattern of the cultivable *Bacillus*-like bacterial communities were soil sampling latitudes (or TC content), but not of soil pH. Moreover, we found that several OTUs with higher relative abundance are the functional or beneficial bacteria, which harbor potential roles in the bioremediation of chromium pollution, extracellular protein production and promotion of plant growth. Thus, the isolation and identification of *Bacillus*-like bacteria are very important for their functional evaluation and application in the future studies.

## Data Availability

Publicly available datasets were analyzed in this study. This data can be found here: https://www.ncbi.nlm.nih.gov/search/all/?term=PRJNA525189.

## Author Contributions

GW and JL conceived the study and wrote the manuscript. JL, GW, and ZG designed the experiments. XC, ZL, ZY, QY, and YS conducted the experiments. JL, ZY, QY, and YS interpreted the results. JJ and XL revised the manuscript.

## Conflict of Interest Statement

The authors declare that the research was conducted in the absence of any commercial or financial relationships that could be construed as a potential conflict of interest.

## Footnotes

1 http://northeast.geodata.cn

2 http://drive5.com/uparse/

3 http://rdp.cme.msu.edu/

## Supplementary Material

FIGURE S1 A map of sampling locations across black soil zone of northeast China.

FIGURE S2 Heatmap profile showing the distribution of the 33 abundant OTUs (relative abundance more than 5% in at least one sample) of *Bacillus*-like bacteria across black soil samples. The abbreviation of the sampling sites was shown in Supplementary Table S1.

FIGURE S3 Principal coordinate analysis of *Bacillus*-like bacterial communities based on the weighted pairwise UniFrac community distances between sites **(A)**. The linear relationships between the PCoA1 score and latitude **(B)**.

TABLE S1 Sample locations and soil physicochemical properties in 26 black soils used in this study.

TABLE S2 Relative abundance (%) of *Bacillus*-like bacterial communities at family and genus levels across all soil samples.

TABLE S3 The linear relationships between PCoA scores and soil total carbon content (TC), and soil pH value. Values in bold indicate significant correlations (*P* < 0.01).
