# Supplementary figures and images for: The Diversity and Geographic Distribution of Cultivable Bacillus-Like Bacteria Across Black Soils of Northeast China
Source: Front Microbiol. 2019 Jun 21;10:1424. doi: 10.3389/fmicb.2019.01424 (PMC6598460; doi:10.3389/fmicb.2019.01424)

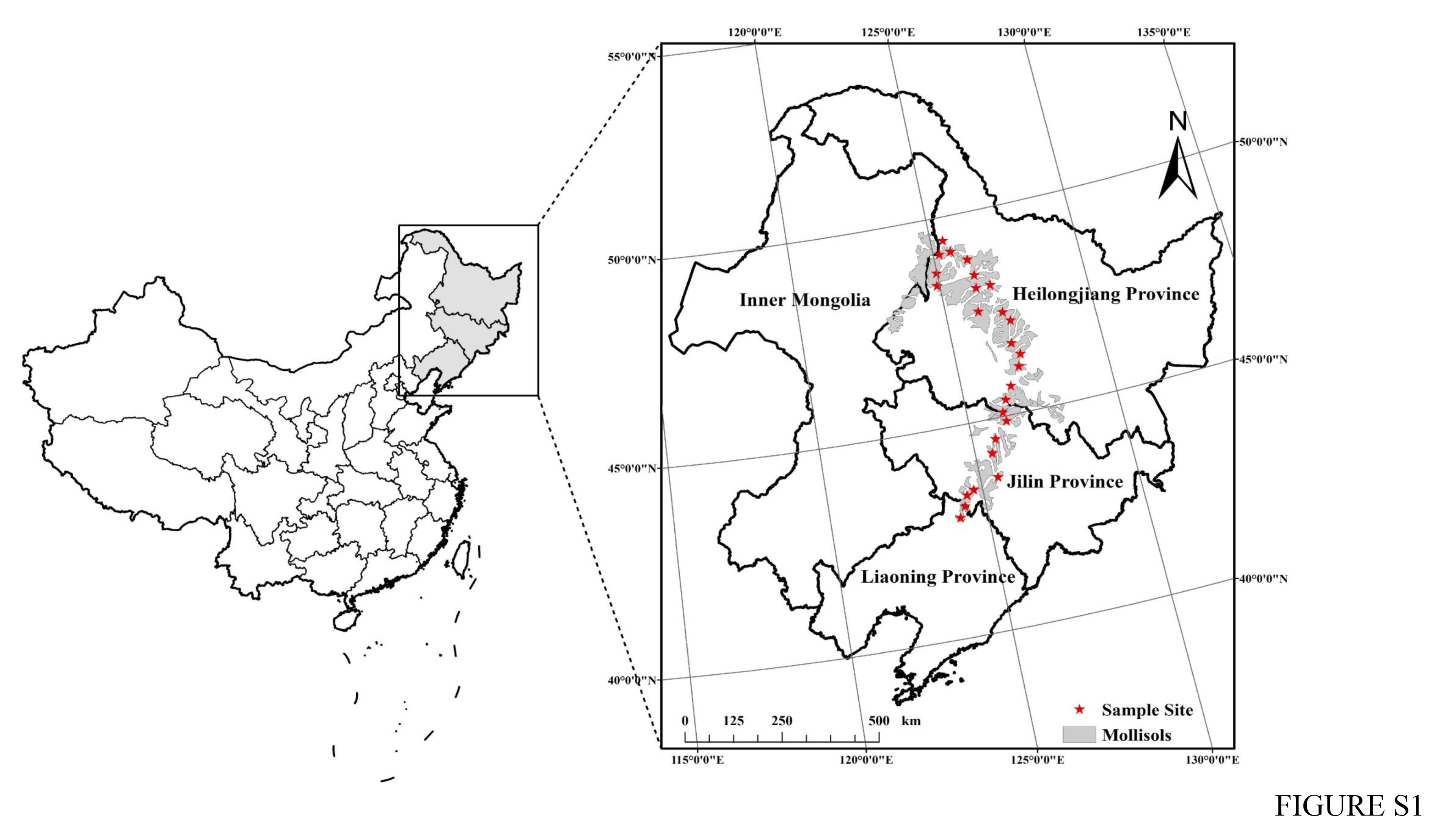

Supplement: FIGURE S1 — A map of sampling locations across black soil zone of northeast China. [file Image_1.JPEG]

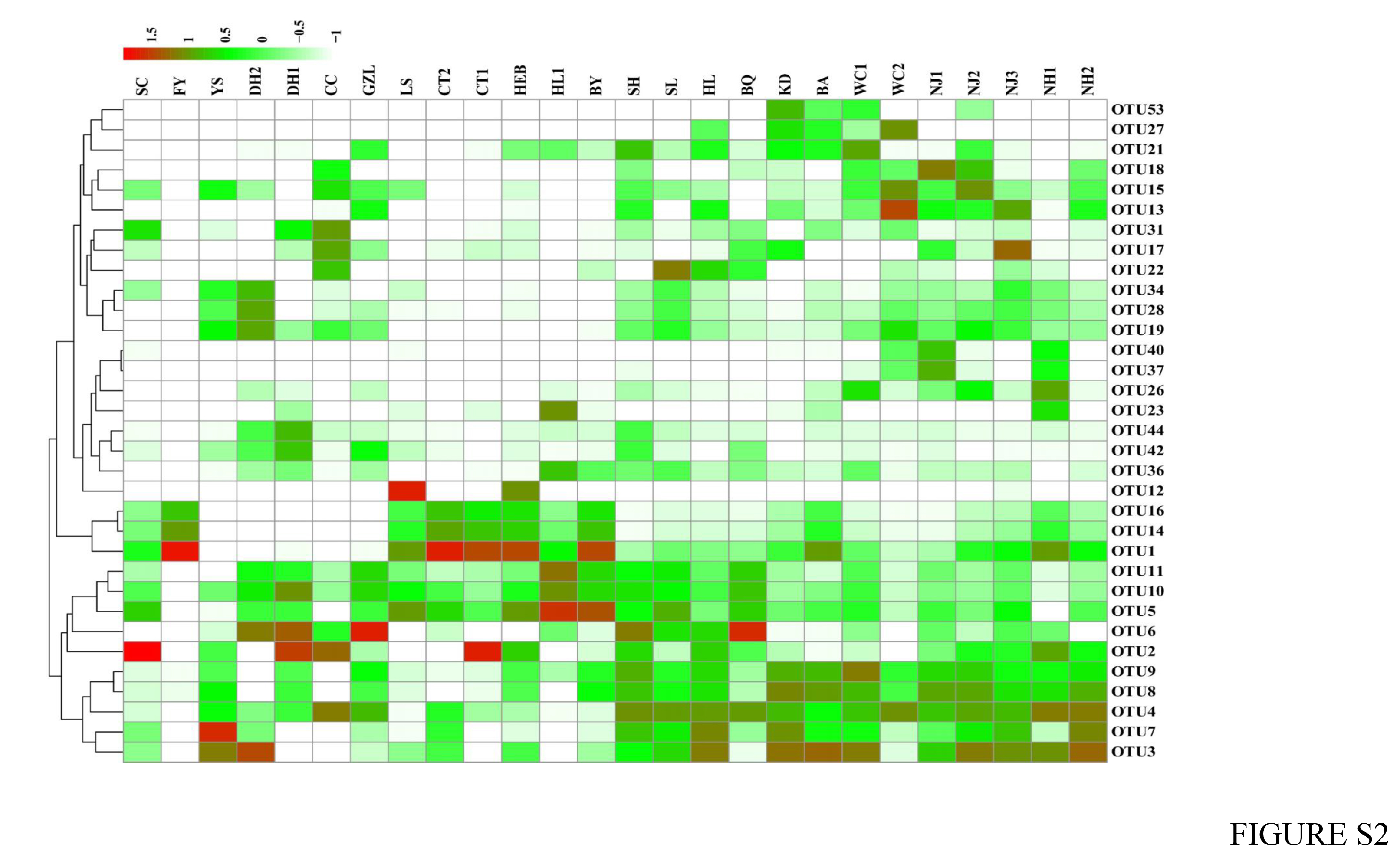

Supplement: FIGURE S2 — Heatmap profile showing the distribution of the 33 abundant OTUs (relative abundance more than 5% in at least one sample) of Bacillus-like bacteria across black soil samples. The abbreviation of the sampling sites was shown in Supplementary Table S1. [file Image_2.JPEG]

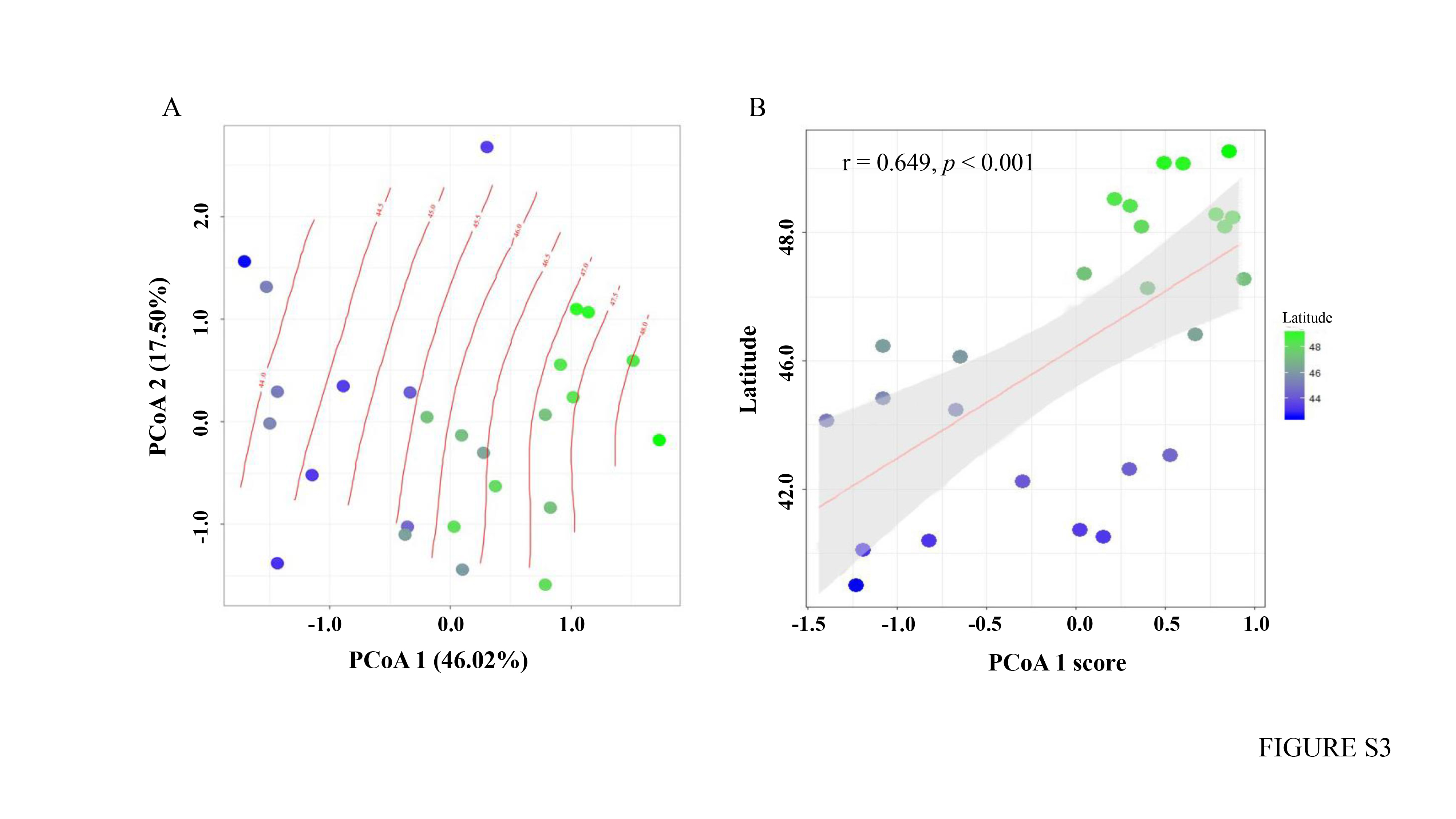

Supplement: FIGURE S3 — Principal coordinate analysis of Bacillus-like bacterial communities based on the weighted pairwise UniFrac community distances between sites (A). The linear relationships between the PCoA1 score and latitude (B). [file Image_3.JPEG]
