# Supplementary material for: The Diversity and Geographic Distribution of Cultivable Bacillus-Like Bacteria Across Black Soils of Northeast China
Source: Front Microbiol. 2019 Jun 21;10:1424. doi: 10.3389/fmicb.2019.01424 (PMC6598460; doi:10.3389/fmicb.2019.01424)
Supplement: TABLE S1 — Sample locations and soil physicochemical properties in 26 black soils used in this study. [file Table_1.DOC]

**Table S1** Sample locations and soil physicochemical properties in 26 black soils used in this study.

| Sample | location | Latitude/Longitude | Crop | pH  (H_2_O) | Total C  (g∙kg^−1^) | Total N  (g∙kg^−1^) | H_2_O  (%) | Total P  (g∙kg^−1^) | Available K  (mg∙kg^−1^) | Available P  (mg∙kg^−1^) | NH_4_^+^-N  (mg∙kg^−1^) | NO_3_^-^-N  (mg∙kg^−1^) |
| --- | --- | --- | --- | --- | --- | --- | --- | --- | --- | --- | --- | --- |
| CT1 | Cangtu 1, Liaoning | 42°50′N/124°07′E | Maize | 5.68 | 14.04 | 1.17 | 22 | 0.90 | 106.9 | 52.00 | 10.24 | 12.16 |
| CT2 | Cangtu 2, Liaoning | 43°05′N/124°20′E | Maize | 5.46 | 14.58 | 1.02 | 21 | 0.63 | 97.2 | 18.50 | 8.87 | 6.71 |
| LS | Lishu, Jilin | 43°20′N/124°28′E | Maize | 6.02 | 11.77 | 0.99 | 25 | 0.65 | 90.6 | 17.00 | 8.98 | 11.66 |
| GZL | Gongzhuling, Jilin | 43°26′N/124°43′E | Maize | 5.50 | 14.40 | 1.12 | 23 | 0.90 | 110.5 | 31.50 | 9.70 | 8.67 |
| CC | Changchun, Jilin | 43°37′N/125°34′E | Maize | 4.95 | 15.59 | 1.26 | 21 | 1.45 | 114.0 | 48.00 | 9.25 | 8.11 |
| DH1 | Dehui 1, Jilin | 44°12′N/125°33′E | Maize | 4.79 | 17.45 | 1.44 | 22 | 0.74 | 110.5 | 40.50 | 9.74 | 13.07 |
| DH2 | Dehui 2, Jilin | 44°31′N/125°45′E | Maize | 4.56 | 14.26 | 1.30 | 22 | 0.80 | 81.5 | 28.00 | 10.77 | 28.47 |
| YS | Yushu, Jilin | 44°53′N/126°14′E | Maize | 5.27 | 20.03 | 1.74 | 22 | 0.81 | 127.8 | 18.50 | 8.75 | 9.01 |
| FY | Fuyu, Jilin | 45°06′N/126°11′E | Maize | 5.78 | 19.97 | 2.03 | 18 | 0.86 | 106.9 | 16.00 | 9.68 | 7.48 |
| SC | Shuangcheng, Heilongjiang | 45°23′N/126°22′E | Maize | 6.53 | 17.02 | 1.68 | 23 | 0.98 | 106.4 | 29.50 | 9.33 | 8.55 |
| HRB | Harbin, Heilongjiang | 45°41′N/126°38′E | Soybean | 6.57 | 26.36 | 1.69 | 23 | 1.40 | 159.9 | 66.50 | 9.61 | 7.53 |
| HL1 | Hulan, Heilongjiang | 46°06′N/127°02′E | Maize | 5.18 | 19.76 | 1.42 | 22 | 0.75 | 96.7 | 17.00 | 10.03 | 9.38 |
| BY | Bayan, Heilongjiang | 46°23′N/127°11′E | Maize | 5.87 | 26.41 | 1.90 | 25 | 1.14 | 169.5 | 50.00 | 11.89 | 18.43 |
| SH | Suihua, Heilongjiang | 46°41′N/126°58′E | Maize | 5.18 | 18.91 | 1.41 | 24 | 0.83 | 114.5 | 28.00 | 10.97 | 10.18 |
| SL | Suiling, Heilongjiang | 47°13′N/127°07′E | Maize | 5.19 | 27.07 | 1.90 | 30 | 0.68 | 97.7 | 38.00 | 13.33 | 9.60 |
| HL | Hailun, Heilongjiang | 47°27′N/126°55′E | Maize | 5.42 | 29.97 | 2.12 | 27 | 1.15 | 106.9 | 25.00 | 10.84 | 6.45 |
| BQ | Baiquan, Heilongjiang | 47°35′N/126°07′E | Maize | 4.98 | 23.41 | 1.95 | 26 | 0.85 | 130.3 | 42.50 | 34.54 | 70.97 |
| KD | Kedong, Heilongjiang | 48°09′N/126°13′E | Soybean | 5.41 | 32.03 | 2.45 | 24 | 1.08 | 102.3 | 31.50 | 9.73 | 20.50 |
| BA | Beian, Heilongjiang | 48°09′N/126°43′E | Soybean | 6.10 | 53.53 | 4.25 | 40 | 1.50 | 151.7 | 36.00 | 13.01 | 16.85 |
| WC1 | Wudalianchi 1, Heilongjiang | 48°28′N/126°15′E | Soybean | 5.43 | 29.92 | 2.36 | 28 | 1.14 | 96.7 | 27.00 | 10.89 | 12.57 |
| WC2 | Wudalianchi 2, Heilongjiang | 48°52′N/126°08′E | Soybean | 5.39 | 36.76 | 3.06 | 35 | 1.33 | 125.7 | 22.00 | 11.12 | 14.06 |
| NH1 | Nehe 1, Heilongjiang | 48°41′N/124°59′E | Maize | 5.35 | 24.78 | 1.93 | 28 | 0.93 | 78.9 | 27.50 | 10.35 | 9.94 |
| NH2 | Nehe 2, Heilongjiang | 48°23′N/124°55′E | Soybean | 5.97 | 23.68 | 1.84 | 25 | 0.87 | 115.1 | 25.00 | 9.80 | 10.60 |
| NJ1 | Nenjiang 1, Heilongjiang | 49°08′N/125°37′E | Maize | 5.53 | 31.71 | 2.50 | 28 | 1.30 | 103.9 | 42.50 | 9.91 | 13.11 |
| NJ2 | Nenjiang 2, Heilongjiang | 49°26′N/125°26′E | Wheat | 5.17 | 37.23 | 2.96 | 33 | 1.23 | 132.4 | 24.50 | 11.74 | 11.00 |
| NJ3 | Nenjiang 3, Heilongjiang | 49°07′N/125°13′E | Soybean | 5.32 | 20.63 | 1.64 | 26 | 0.96 | 122.2 | 42.00 | 10.00 | 9.46 |
