# Supplementary material for: The Diversity and Geographic Distribution of Cultivable Bacillus-Like Bacteria Across Black Soils of Northeast China
Source: Front Microbiol. 2019 Jun 21;10:1424. doi: 10.3389/fmicb.2019.01424 (PMC6598460; doi:10.3389/fmicb.2019.01424)
Supplement: TABLE S3 — The linear relationships between PCoA scores and soil total carbon content (TC), and soil pH value. Values in bold indicate significant correlations (P < 0.01). [file Table_3.DOCX]

Table S3 The linear relationships between PCoA scores and soil total carbon content (TC), and soil pH value. Values in bold indicate significant correlations (*P* < 0.01).

| Soil parameter | Weighted | | | | |  | Unweighted | | | | |
| --- | --- | --- | --- | --- | --- | --- | --- | --- | --- | --- | --- |
|  | PCoA1 | |  | PCoA2 | |  | PCoA1 | |  | PCoA2 | |
|  | r | *p* |  | r | *p* |  | r | *p* |  | r | *p* |
| pH | -0.321 | 0.113 |  | -0.062 | 0.765 |  | -0.215 | 0.290 |  | -0.301 | 0.103 |
| TC | **0.601** | <0.001 |  | -0.212 | 0.298 |  | **0.513** | 0.007 |  | -0.130 | 0.525 |
